# Supplementary material for: Crop wild relative populations of Beta vulgaris allow direct mapping of agronomically important genes
Source: Nat Commun. 2017 Jun 6;8:15708. doi: 10.1038/ncomms15708 (PMC5467160; doi:10.1038/ncomms15708)
Supplement: Supplementary Information — Supplementary Figures and Supplementary Tables [file ncomms15708-s1.pdf]

Supplementary Figure 1

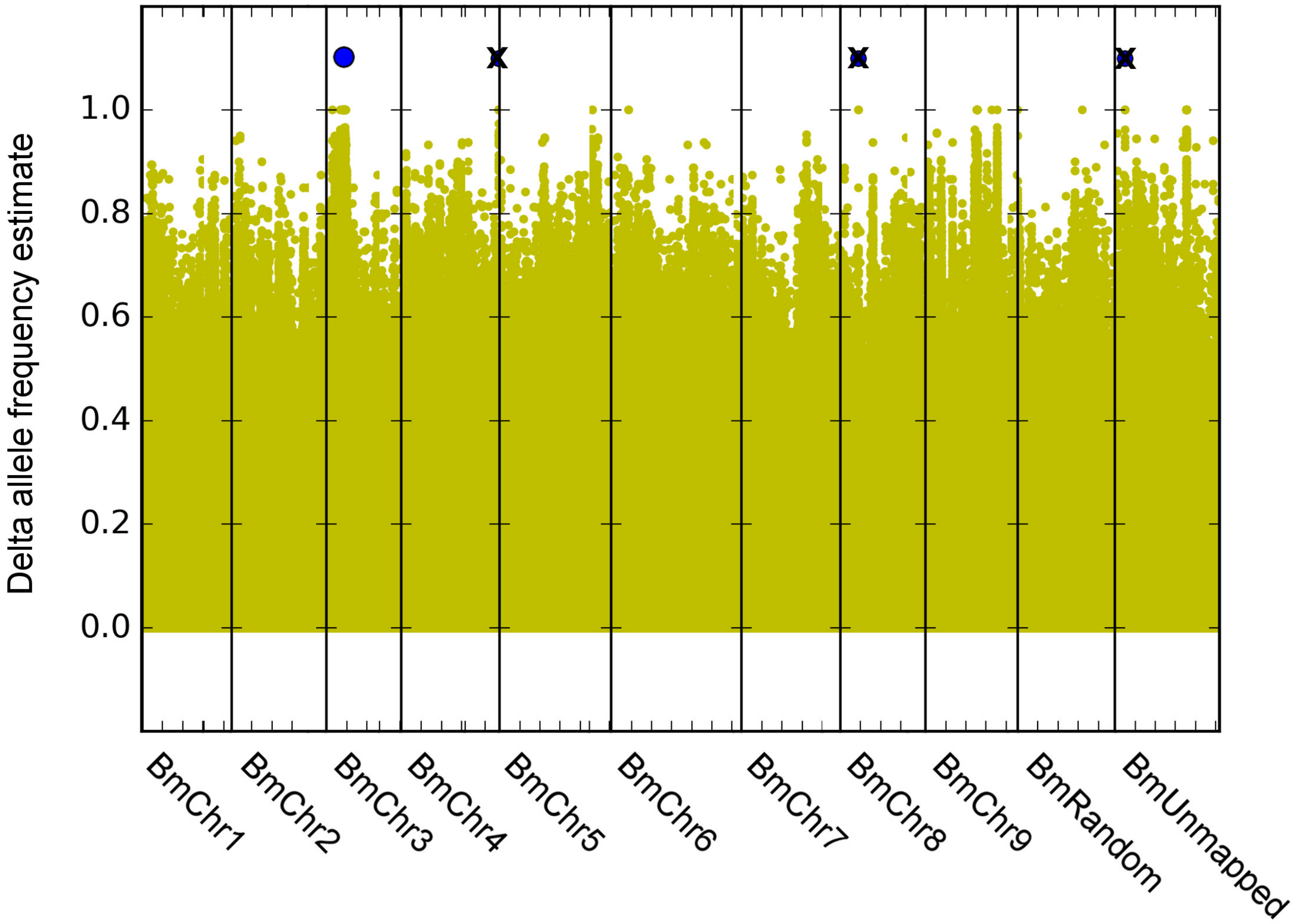

Plot of delta-AFe of sequence variants detected between resistant and susceptible pool over the genome sequence of the WB42 line of *B. vulgaris* ssp. *maritima*.

The delta-AFe values for all detected variants (yellow dots) were plotted along the reference sequence WB42-v2 which has been concatenated and assembled into pseudochromosomes (BmChr1 to 9); BmRandom represents the WB42 scaffolds that map to the unassinged fraction of RefBeet-1.2, and BmUnmapped those WB42 scaffolds that do not map to RefBeet-1.2. On the left are the upper ends of *B. vulgaris* chromosomes according to standard orientation. BmChr3 shows a clear peak. Genomic intervals with highly significant separation of sequence variants between the two pools are indicated by blue circles. Due to the resolution of the plot, the different sizes of the intervals are not visualized (see **Supplementary Figure 2**). Four intervals adding up to 552 kbp in a region of 720 kbp in length are represented by the circle marking BmChr3, and 93 kbp are indicated by the remaining circles on three other pseudochromosomes (see **Supplementary Table 5**). These three loci (marked with 'X') were addressed by mapping, and turned out to be not closely linked to the resistance phenotype (see **Supplementary Table 6**).

Supplementary Figure 2

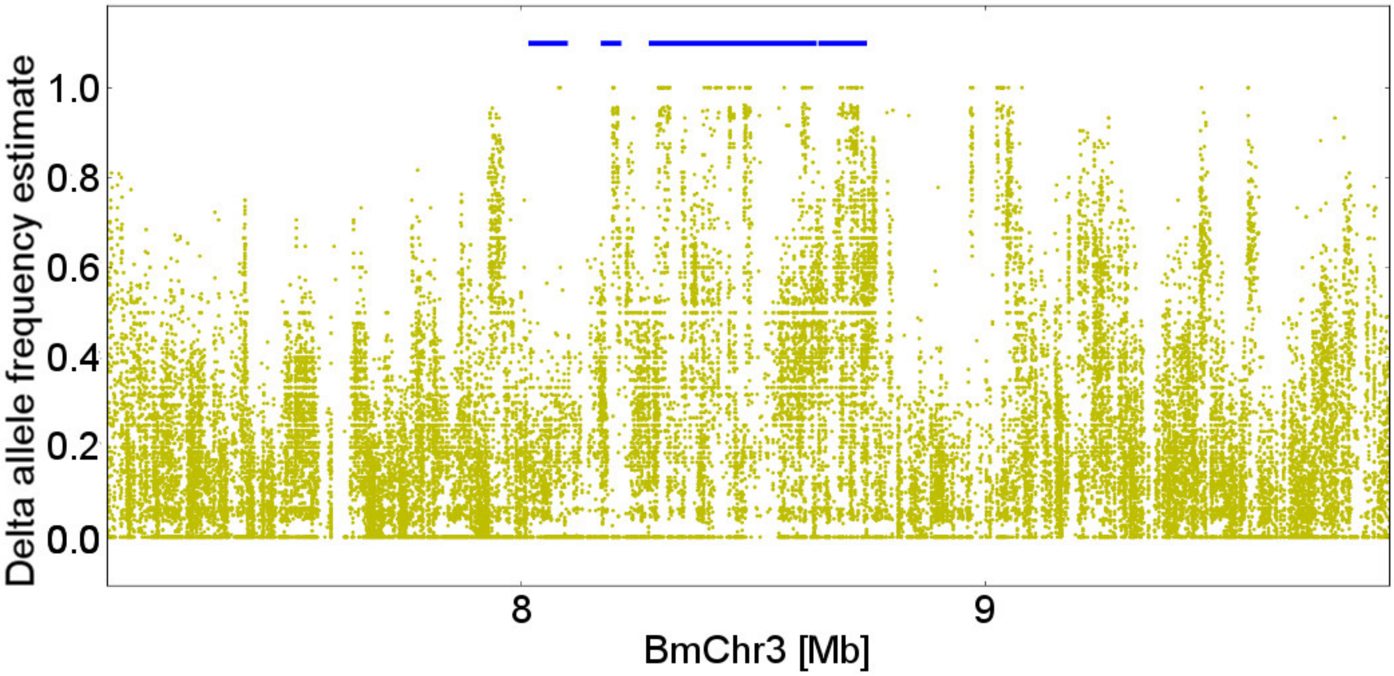

Plot of delta-AFe values zoomed into the genome region on BmChr3 containing the most significant cluster of genetic intervals.
The genetic intervals shown (blue lines) were detected by an algorithm described in Methods. Design as described for **Supplementary Figure 1**.

Supplementary Figure 3

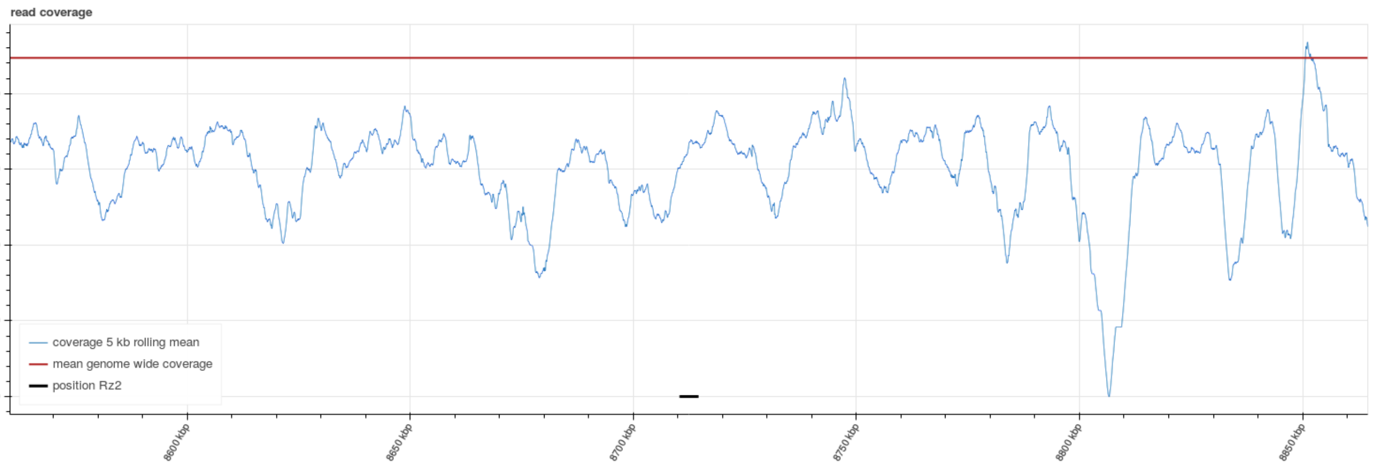

Read coverage plot of the *Rz2* locus.
A region of in total about 300 kbp upstream and downstream of *Rz2* is visualized. The position of the *Rz2* gene is marked with a black line (botton of the plot). Reads generated from the susceptible and the resistant MBS pool were combined and mapped to the WB42-v2 assembly. The mean coverage value of all positions within a 5 kbp window is indicated by a blue line. The red line shows the genome wide mean coverage value.

Supplementary Figure 4

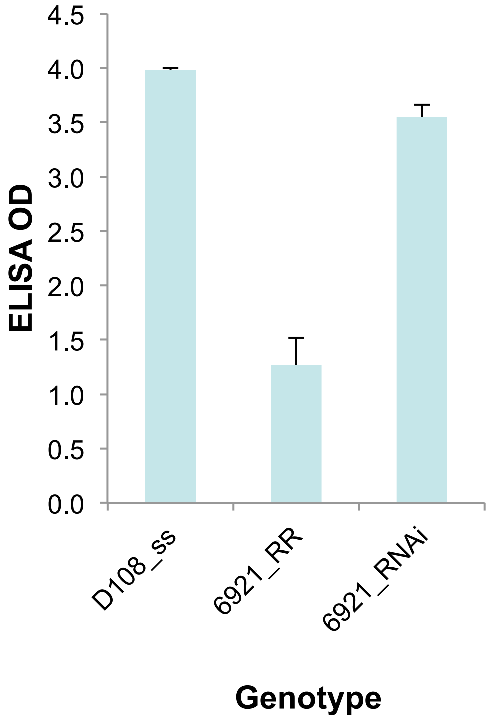

ELISA optical density (ELISA OD) readings of the rhizomania susceptible line (D108_ss, N=8) the resistant line (6921_RR, N=25) and the RNAi transformed resistant line with a dsRNA directed against *Bv3_jumg* (6921_RNAi) that all were tested in a greenhouse assay (see Material). Error bars indicate the standard error of the mean.

Supplementary Table 1: Hardy-Weinberg-Equilibrium (HWE) analysis of markers associated to rhizomania resistance.

Marker	Allele 1[a]		Heterozygous		Allele 2[b]		Allele frequency	χ^{2c}	*P* value[d]
	obs.	exp.	obs.	exp.	obs.	exp.			
**CAU3870**	63	64.95	95	91.10	30	31.95	0.59	0.34	0.56
**CAU3871**	43	44.30	97	94.40	49	50.30	0.48	0.14	0.71
**CAU3872**	16	21.67	96	84.66	77	82.67	0.34	3.39	0.07
**CAU3873**	14	14.73	70	68.54	79	79.73	0.30	0.07	0.79
**CAU3874**	8	6.78	55	57.45	123	121.78	0.19	0.34	0.56
**CAU3875**	94	6.38	76	34.35	11	46.27	0.27	61.87	4.00E-15
**CAU3876**	24	11.94	47	71.12	118	105.94	0.25	21.74	3.00E-06
**CAU3877**	58	39.80	49	85.39	64	45.80	0.48	31.06	3.00E-08
**CAU3878**	18	12.72	57	67.56	95	89.72	0.27	4.15	0.04
**CAU3879**	27	27.19	89	88.61	72	72.19	0.38	0.01	0.95
**CAU3880**	9	11.94	77	71.12	103	105.94	0.25	1.29	0.26
**CAU3881**	58	38.14	52	91.72	75	55.14	0.45	34.69	4.00E-09
**CAU3882**	8	11.94	79	71.12	102	105.94	0.25	2.32	0.13
**CAU3883**	9	8.72	61	61.57	109	108.72	0.22	0.02	0.90
**CAU3884**	9	9.76	67	65.47	109	109.76	0.23	0.10	0.75
**CAU3885**	14	11.81	66	70.37	107	104.81	0.25	0.72	0.40
**CAU3886**	43	30.49	55	80.01	65	52.49	0.43	15.93	7.00E-05
**CAU3887**	34	14.86	38	76.27	117	97.86	0.28	47.59	5.00E-12
**CAU4188**	6	12.05	75	62.89	76	82.05	0.28	5.82	0.02

[a] allele 1, observed in the reference sequence WB42;

[b] allele 2, an alternative allele when compared to the reference sequence WB42;

[c] χ^2 for deviation from HWE;

[c] *P* value for χ^2

Supplementary Table 2: Illumina read data of phenotypic pool sequencing and coverage of the concatenated WB42-v2 genome sequence.

**Sample**	**Read length**[a]	**Insert size**[b]	**Depth of coverage**			
			**Untrimmed**[c]	**Trimmed**[d]	**Mapped**[e]	**Reference covered**[f]
R1/B2444[g]	101	356	42.2	23.3	22.4	59.8
S1/B2446[g]	101	346	39.9	23.2	22.3	57.8

[a] Length of the sequenced reads in bp, all generated as paired ends (PE);
[b] average length of the PE sequenced fragments in bp;
[c] theoretical mean number of untrimmed reads covering each base of WB42-v2;
[d] theoretical mean number of trimmed reads covering each base of WB42-v2;
[e] mean number of uniquely mapped reads covering each base of WB42-v2;
[f] percentage of the WB42-v2 sequence covered with at least 15 uniquely mapped reads;
[g] R1 represents the resistant pool, S1 the susceptible pool.

Supplementary Table 3: WB42-v0 assembly details based on scaffolds and contigs larger than 500 bp.

Total size (bp)	Number of sequences	N50 size (bp)	N50 number	N90 size (bp)	N90 number	Average length (bp)	Largest scaffold (bp)
531,940,822	57,361	59,342	2,449	6,828	11,696	9,274	899,438

Supplementary Table 4: Relevant genetic intervals identified by MBS between the resistant pool R1 and the susceptible pool S1.

Interval	Region	Start[a]	End[a]	Size[c] [bp]
Interval 1	BmChr3[b]	8,022,404	8,096,342	73,939
Interval 2	BmChr3[b]	8,179,036	8,210,757	31,722
Interval 3	BmChr3[b]	8,280,341	8,632,606	352,266
Interval 4	BmChr3[b]	8,647,027	8,741,209	94,183
Interval 5	BmChr4Un	16,782,347	16,801,677	19,331
Interval 6	BmChr4Un	16,805,004	16,817,848	12,845
Interval 7	BmChr8	8,873,963	8,905,087	31,125
Interval 8	BmUnmapped	4,989,906	5,020,361	30,456

[a] Start and end position from automatic interval detection in the WB42-v2 genome assembly;
[b] four closely connected intervals in BmChr3 covering a genome region of 0.72 Mbp (target region);
[c] note that the target region has a size of about 0.72 Mbp and overlaps with 25 scaffolds of WB42-v2, while the 4 closely connected intervals on BmChr3 overlap with 23 scaffolds of WB42-v2.

Supplementary Table 5: Phenotype and marker analysis results of selected genotypes of the Kalundborg population.

		Marker [a]			
Genotype	Phenotype	CAU3880	CAU4220	CAU4221	CAU4222
091016	Resistant	A	B	A	H
091055	Resistant	A	B	A	B
091101	Susceptible	B	A	B	A
091134	Susceptible	B	A	A	A
091195	Susceptible	B	A	H	A
091239	Resistant	A	A	B	A
091279	Susceptible	B	A	H	A
091283	Susceptible	B	H	A	A
091291	Resistant	A	B	A	B
091343	Resistant	A	B	B	B

[a] A, allele observed in the WB42 sequence; B, an alternative allele when compared to the WB42 sequence; H, heterozygous

Primer sequences, SNP positions, and variant positions for the Kalundborg population.

Marker	Primers[a]	Length (bp)	Region	Position	WB42 allele[b]	Alt. allele[c]
CAU4220	GATGCACCATGTACCCCTCT<br>TTCGTTAATGCTATTTCTGGATGA	430	BmUnmapped Interval 8	4,990,130	C	T
CAU4221	GCGAGGAACACGAGAGAAAG<br>TCACTACCCCACGAATGTTG	322	BmChr8 Interval 7	8,884,673	C	A
CAU4222	GCGAGGAACACGAGAGAAAG<br>TCACTACCCCACGAATGTTG	747	BmChr4Un[d] Intervals 5 & 6	16,789,484	C	T

[a] Sequence from 5' to 3';

[b] Allele observed in the WB42 sequence;

[c] Alternative allele when compared to the WB42 sequence;

[d] Note that this target region two different intervals have been identified, both located in one scaffolds of WB42-v2.

Supplementary Table 6: SNP region used for KASP marker development

CAU marker	Sequence	WB42 allele	alt_ allele	Position in BmChr3
**3870**	TTTTCTAAGATGTCGGCTCACTATAAAATT[G/A]GGTGGACTCTATCAATAAAAAAATTAGTTT	G	A	8,204,769
**3871**	TATAGGGATAAGTTATGGGTTTTTCGAGAT[C/T]TATTTAGAAAATAGAGGAGTGTCTTAGGGA	C	T	8,243,424
**3872**	ACAAAATTGAAGTAATATTTTAGACCTTTT[A/G]GTTTTCCGCTAGTTCTTTCTCTACSCACTT	A	G	8,199,959
**3873**	AAAGTAAAGCATTTTGGAAGGTCAAGTTGA[C/T]GATCCATACTTCAATCTATTTTTGTCGCTC	C	T	8,198,636
**3874**	GGAAGCCCACCTCCTCTTTCACCAATGACT[C/T]TAGCAATTCTACGTACAGCTGAAATATTAG	C	T	8,481,152
**3875**	AAGCAGTWAGTGCCACTACAATATTAAGAA[C/A]TTGCTCTTATAAATAGGTAATTGGAAATTA	C	A	8,614,134
**3876**	TGACCATGAGACATTTGGGTCGGGTTAGAA[A/G]TACRCCTATAATACAAAATTAATTAAATAT	A	G	8,473,154
**3877**	ACAACCTCAAAGTACTTTACTAAAAGAATT[G/A]CAATTGTTCACAACAAGTTCTCATTTTTTC	G	A	8,485,068
**3878**	AGCAATRCAGTCGGCTGCTCTATTGACTTC[T/-]TCTGAAGACATGTCGTACATGATAAGAGTG	T	-	8,492,848
**3879**	AGATTCACTAGAAAATTCAAATTATCAAGA[T/C]ATCTCTTCTAGTCTTATCTGATGCKGGCCA	T	C	8,606,234
**3880**	CCCTCCRAACYCTTTCAATRGTGCATTGCA[C/G]TAAACTGSAATCAGTGCCAGATTGGATGCC	C	G	8,713,878
**3881**	AAGGTCATCCAACTCAATAATGTTCTAGGG[T/C]GAGAGCCGGGTACCACTCTTATCAGTGAAC	C	T	8,706,636
**3882**	GAAGCTTTGAATTACTGATATGYTGTAGTT[T/G]GGTACTTGGTTTGATCTGTGAGATTATTCA	T	G	8,722,130
**3883**	AACTTTTGGCACTCATATCTGAAGAAGAAG[A/G]TTGCTAAGACAGAAAAAATyAArCCCATCT	A	G	8,459,518
**3884**	ATAAAAATTAATGGCTCATATATTCTCTTT[T/C]CAATATTTTCCTCCCTCCTAATACTAAGAA	T	C	8,486,370
**3885**	GGGTCCAYATCCATCTCATCGTCCTCGTCG[G/T]CATCATGATCATCGTGCACTTCATGACCAT	G	T	8,321,187
**3886**	ATTATAAGAGATTTCGTTGAATTCAAGTTC[A/T]CATAGCTTAAGTAGGAGAAAAATAATCTTT	A	T	8,418,708
**3887**	TCATAGCTAAGATCCCTAATGATCCCAGTC[C/A]TCTYTTCCCTGCCCACAAGTTTCGKCTAAG	C	A	8,426,426
**4072**	ATAAATTTGACCCTAACCCTCTAGTTATTG[A/G]GCTACCCTGTCCAATAACCAAGTCTACTAA	A	G	8,013,930
**4074**	AGAAAACTTGAACTGGTTTCAAAGAAGGAA[G/T]TGCCGGGTATATGATAACCATATCAAAGAC	G	T	8,181,247
**4075**	TGAAGTCTTGTTAGATTCGAATCAATGCGA[G/T]AATTTCAAATATCAAATTTCTATAGTTTTT	G	T	8,155,956
**4076**	TTACCTATTRATTTCCACCTTCCTCACATT[C/A]CACGTTGCAGCGCAAAGATACAATAGGCAA	C	A	8,228,009
**4077**	TAACAGCTTTAAAGGAAGGCAGTTTAGTGG[G/A]AAGTTYAATAATGTCCCAGCAGACCAACAT	G	A	8,237,302
**4078**	TCACTGCGTCTGTTTTTCTCTTCAGTAGGG[C/A]TTGAAATGTAGCTCGATCATAAGGAGGCGG	C	A	8,252,760
**4079**	CATCATCTCAGAAAATTGACGAATATATCC[T/C]GATTTACTTGATTTATTCACAAAATTACCA	T	C	8,260,131
**4082**	TCTGATTTCATTTCAGGATAAAAGTCTATC[G/A]AGTGGGATATTTTTCCTGGAACTTCTTAGT	G	A	8,526,802
**4083**	GAGTTTTCTTTTCTCCTAGATTTAGGTTAC[TGT/-]TTCCATGCAATTTTCTTCCTGATTATCTCC	-	TGT	8,568,151
**4084**	TTCACACAAACAACTAGTCAGACTAAACCA[G/A]CTAATAACTAATTTAAACATCKAACCTCTA	G	A	8,571,825
**4085**	AGTAGCACCATTGCAAAAGTGCACTTCTCA[T/A]TGTTTCTTGGTCATGTGCCAATTTCTCCAA	T	A	8,597,683
**4086**	GGTTGATTTGATTGAATTTTGTGCGGCGTT[G/A]CAACAGATGTCAGTATTTTGTTTACAATCA	G	A	8,622,221
**4088**	AGGTGGATAAAAAGAATGGGATGGTATGAT[T/A]TTTTGACTTTGCACTACTATTTGGTTAATM	T	A	8,694,501
**4089**	GTTTGCTTTCTTCGGCATTGCTAGTGCTCT[T/C]AAAGCTTCTGAAGGTGATGCTGATCTAATT	T	C	8,760,088

Supplementary Table 7: Association analysis results of the Kalundborg Population after evaluation of 189 families tested in 4 independent greenhouse rhizomania resistance tests.

**Marker**[a]	**Test 1**[b]		**Test 2**		**Test 3**		**Test 4**	
	**p-value**[c]	**r^2**	**p-value**	**r^2**	**p-value**	**r^2**	**p-value**	**r^2**
**CAU3880**	1.70E-17	0.36	5.15E-12	0.24	3.64E-14	0.28	5.82E-10	0.20
**CAU3882**	7.83E-16	0.34	3.34E-11	0.24	2.86E-11	0.24	1.22E-07	0.17
**CAU4188**	2.57E-12	0.28	6.11E-08	0.18	5.33E-10	0.22	5.41E-07	0.16
**CAU3874**	9.01E-11	0.25	1.46E-07	0.17	2.28E-09	0.21	1.60E-06	0.15
**CAU3875**	1.78E-09	0.22	7.56E-07	0.16	1.02E-08	0.19	3.70E-05	0.12
**CAU3870**	4.68E-10	0.22	2.01E-05	0.12	5.62E-15	0.31	1.08E-06	0.15
**CAU3884**	2.84E-09	0.22	1.71E-06	0.15	1.01E-05	0.13	1.76E-05	0.12
**CAU3883**	3.16E-09	0.22	9.37E-06	0.13	ns[d]	ns	6.58E-05	0.11
**CAU3876**	1.38E-07	0.16	1.49E-07	0.16	2.05E-07	0.15	1.77E-05	0.11
**CAU3872**	8.87E-07	0.16	5.99E-08	0.18	7.88E-04	0.09	3.79E-04	0.09
**CAU3886**	2.31E-06	0.15	1.37E-06	0.15	2.42E-05	0.12	ns	ns
**CAU3879**	6.82E-06	0.14	8.62E-05	0.11	4.70E-06	0.14	ns	ns
**CAU3885**	1.19E-05	0.14	5.97E-04	0.09	1.16E-04	0.11	ns	ns
**CAU3887**	4.78E-06	0.13	3.76E-06	0.13	6.13E-06	0.12	8.80E-07	0.14
**CAU3878**	2.32E-05	0.13	ns	ns	5.60E-04	0.09	ns	ns
**CAU3873**	5.80E-05	0.12	9.46E-04	0.08	ns	ns	ns	ns
**CAU3877**	1.62E-04	0.11	2.47E-04	0.10	1.97E-06	0.15	3.26E-06	0.14
**CAU3871**	5.79E-05	0.10	1.10E-04	0.09	ns	ns	ns	ns
**CAU3881**	2.62E-04	0.10	1.44E-05	0.13	ns	ns	ns	ns

[a] Only markers with positive association to resistance in at least 2 tests are shown;
[b] markers are ordered according to their r^2 in test 1;
[c] the P values are derived from single marker F-test;
[d] the result is not statistically significant

Supplementary Table 8: Sugar beet breeding material used in the validation panel of *Rz2* markers.

**Company**	**Rhizomania resistant**			**Rhizomania susceptible**
	Rz1	***Rz2***	***Rz1+Rz2***	
**KWS**	55	10	2	27
**Syngenta**	31	7	6	47
**SESvanderHAVE**	66	5	3	20
**Total**	97	22	11	149

Supplementary Table 9: Evaluation of deduced amino acid sequences of Bv3_jumg alleles across a panel of resistant and susceptible plants from the Kalundborg population and sequenced susceptible sugar beet genotypes.

Plant material	Rhizomania phenotype	Aminoacid position (a)																																																	
		35	149	233	245	259	263	265	290	299	300	301	314	347	354	393	395	406	420	428	438	472	479	481	485	518	544	568	572	582	646	661	684	688	709	720	777	793	800	802	806	811	831	834	836	837	855	862	870	874	887
Resistant_Kalundborg	R	R	M	Q	A	Q	Q	R	D	R	S	R	A	A	I	D	G	P	K	V	Q	R	V	A	G	S	C	Y	M	L	H	L	C	L	K	T	V	F	L	N	L	H	S	N	R	C	R	C	G	G	T
Susceptible_Kalundborg	S	Q	I	Q	S	Q	Stop	-	-	-	-	-	-	-	-	-	-	-	-	-	-	-	-	-	-	-	-	-	-	-	-	-	-	-	-	-	-	-	-	-	-	-	-	-	-	-	-	-	-	-	-
YMoBv	S	Q	I	Q	S	Q	Stop	-	-	-	-	-	-	-	-	-	-	-	-	-	-	-	-	-	-	-	-	-	-	-	-	-	-	-	-	-	-	-	-	-	-	-	-	-	-	-	-	-	-	-	-
YTiBv	S	Q	I	K	S	H	Q	R	Y	Q	R	G	A	P	V	C	A	P	R	M	K	Q	I	T	Y	N	Y	C	L	V	Y	M	W	Q	E	K	A	Y	S	C	M	C	T	N	S	F	L	H	R	G	Transposon insertion
RefBeet1.2	S	Q	I	K	S	H	Q	R	Y	Q	R	G	A	P	V	C	A	P	R	M	K	Q	I	T	Y	N	Y	C	L	V	Y	M	W	Q	E	K	A	Y	S	C	M	C	T	N	S	F	L	H	R	G	Transposon insertion
KDHBv	S	Q	I	K	S	H	Q	R	Y	Q	R	G	A	P	V	C	A	P	R	M	K	Q	I	T	Y	N	Y	C	L	V	Y	M	W	Q	E	K	A	Y	S	C	M	C	T	N	S	F	L	H	R	G	Transposon insertion
UMSBv	S	Q	I	K	S	H	Q	Q	D	Q	R	G	T	P	V	C	A	L	R	M	K	Q	I	T	Y	N	Y	C	L	V	Y	M	W	Q	E	K	A	Y	S	C	L	C	T	I	S	F	L	H	R	Transposon insertion	-

(a) Positions of changed amino acids is given relative to the first amino acid (translation start) of the protein encoded by Rz2_KD-R (Bv3_jumg).
